# A Case–Control Study of Tackle-Based Head Injury Assessment (HIA) Risk Factors in the National Rugby League

**DOI:** 10.1186/s40798-021-00377-9

**Published:** 2021-11-17

**Authors:** Andrew J. Gardner, Grant L. Iverson, Suzi Edwards, Ross Tucker

**Affiliations:** 1 Priority Research Centre for Stroke and Brain Injury, School of Medicine and Public Health, University of Newcastle, Callaghan, NSW Australia; 2 Department of Physical Medicine and Rehabilitation, Harvard Medical School, Boston, MA USA; 3 Spaulding Rehabilitation Hospital and Spaulding Research Institute, Charlestown, MA USA; 4 MassGeneral Hospital for Children Sports Concussion Program, Boston, MA USA; 5 Discipline of Exercise and Sport Science, The University of Sydney, Camperdown, NSW Australia; 6 Priority Research Centre for Stroke and Brain Injury, University of Newcastle, Callaghan, NSW Australia; 7 World Rugby, Pty (Ltd), Dublin, Ireland

**Keywords:** Tackle, Injury prevention, Head impact events, Head injury assessment, Concussion

## Abstract

**Background:**

The tackle is the in-game activity carrying the greatest risk for concussion in rugby. A recent evaluation of tackle characteristics in rugby union precipitated a rule modification to reduce head impact risk during tackles. This study aims to replicate the work conducted in rugby union by examining the association between tackle characteristics and head injury events in professional rugby league.

**Methods:**

There were 446 tackles resulting in a head injury assessment (HIA) and 5,694 tackles that did not result in a head injury from two National Rugby League (NRL) seasons that were reviewed and coded. Tackle height, body position of players, and contact area on an opponent’s body were evaluated, with the propensity of each situation to cause an HIA calculated as HIAs per 1000 events.

**Results:**

The propensity for tacklers to sustain a head injury was 0.99 HIAs per 1000 tackles, 1.74-fold greater than for the ball carrier (0.57 HIAs per 1000 tackles). There was a 3.2-fold higher risk for an HIA when the tackler was upright compared to bent-at-the-waist. The greatest risk of a tackler HIA occurred when head contact was very low (knee, boot) or high (head and elbow). HIAs were most common following head-to-head impacts. The lowest propensity for tackler HIA was found when the tackler’s head was in proximity with the ball carrier’s torso.

**Conclusions:**

The result of this study replicated the findings in professional rugby union. This has implications for the injury prevention initiatives implemented to reduce HIA risk because the majority of injuries are sustained by the tackler.

**Supplementary Information:**

The online version contains supplementary material available at 10.1186/s40798-021-00377-9.

## Key Points

- The tackler is more likely to sustain a head injury than the ball carrier.
- Upright tackles result in a higher proportion of head-to-head contact.
- The HIA risk is lowest when the tackler and the ball carrier are both bent in the tackle.
- Injury prevention initiatives aimed at reducing the tackle height (tackle technique modification) might reduce the HIA risk.

## Introduction

Rugby league is an international full-contact collision sport that involves numerous tackle events. There is a large body of work that has investigated rugby league tackle proficiency as it relates to player and team performance [1–4], but another important aspect of evaluating the tackle pertains to injury risk [5–11]. The tackle is the rugby league game event identified as causing the most injuries [12–15]. Specifically, the tackle has been identified as the game event most commonly associated with clinically diagnosed concussion in professional rugby league [16, 17]. Video analysis of concussion in rugby league [18–22] has revealed that the tackler is more frequently concussed than the ball carrier, and that head-to-head and head-to-shoulder contact in upper body tackles, and head-to-hip contact in lower body tackles are the most frequent mechanism for head impact events in concussed tacklers [18–23]. This body of work was primarily concerned with identifying the signs of concussion through video analysis, and did not provide a detailed mechanistic description of the contributing risk factors to the head injury, which meant that propensity and relative risk of various player behaviours was not a primary outcome. In addition, the sample sizes of these studies were comparatively small and one of the studies was focused on youth, not adult rugby league players. In professional rugby union (a full-contact collision sport similar to rugby league), (1) upright tacklers with higher contacts on the ball carrier’s body, (2) tackler speed, and (3) acceleration have been found to result in greater propensity for head injury events [24, 25] and concussions [26]. Consideration of the tacklers’ and ball carrier’s body positions, nature of head contact, and the ball carrier’s evasion method are all variables that may play a role in modifying the risks for head injury [26]. In particular, the finding that upright body position and higher contact tackles were more likely to cause Head Injury Assessments (HIAs) drove injury prevention initiatives including emphasis on current tackle height laws and potential law changes in an attempt to create more tackles where the tackler adopts a bent at the waist body position (i.e. thorax segment hinged at the waist, independent of knee or hip posture) and avoids tackles where head-to-head contact is likely [27]. Given the findings regarding head injury risk during tackles in professional rugby union, and the game play similarities between the two rugby codes, the current study sought to replicate the rugby union studies in professional rugby league. The primary objective of this study was to review and code video footage of tackles that resulted in HIAs, medically diagnosed concussions, and a series of tackles that did not result in any head impact, to explore specifically how the body position of the tackler and ball carrier was associated with the propensity and incidence of HIAs to both players during tackles in the National Rugby League.

## Methods

### Participants

This case–control study was conducted in the National Rugby League over two seasons (2017 and 2018). The NRL includes sixteen clubs that compete over a 24-game season and a four-week postseason between eight qualifying teams. Therefore, during the regular season, there are 192 regular season matches (8 pairs of teams, each playing in 24 games) and nine playoff matches, resulting in a total of 402 matches in the study cohort. In accordance with the NRL and Rugby League Players Association Collective Bargaining Agreement, all athletes consented a priori to have their deidentified injury data used in research endorsed by the Rugby League Research Committee. The study was endorsed by the Rugby League Research Committee and approved by the institution human ethics committee and the study was performed in accordance with the standards of ethics outlined in the Declaration of Helsinki.

### Procedures

Head impact events (HIEs) were detected through the NRL’s in-game injury surveillance system. For the 2017 and 2018 seasons there were two levels of in-game injury surveillance: (1) a sideline injury surveillance (SIS) system and (2) the team medical staff. An HIE is defined as a clear head impact sustained by a player, and which is then monitored by the sideline medical staff or matchday video reviewer for possible clinical follow-up in the form of a Head Injury Assessment (HIA). All HIEs are thus identified by either the (1) SIS, or (2) team doctor, were recorded and uploaded to the *GamePlan* Application. A HIA was identified as a head impact event that necessitated either the permanent removal from the game of a player with a confirmed concussion, or the temporary removal of a player with a suspected concussion for an off-field head injury evaluation, as per the NRL concussion recognition and management process. HIEs and HIAs thus differ with respect to the clinical outcome or action taken after the impact is observed.

For the present study, HIAs were selected as the criteria for a head injury case because these are discrete events that are sufficient to cause a player to be removed from play with suspected or confirmed concussion, in large enough numbers to power the analysis. The HIAs also have implications for match-play because they cause either the permanent removal of a player with a concussion from the game, or a fifteen-minute temporary interchange during which time the player undergoes the off-field evaluation. It is thus relevant for the sport to assess what factors contribute to these in-game disruptions.

Video footage of HIAs was clipped and loaded on the *GamePlan* Application. The first author was provided access to the NRL’s *GamePlan* Application subscription and all video clips by the NRL. Most clips provided multiple camera angles of the event, in normal speed and in slow motion. The first author reviewed all video clips and coded all variables, in accordance with the procedure applied by Tucker and colleagues [24, 25], using a predefined coding matrix (Table 1, Additional file 1: Supplementary material). The coding matrix comprised 36 categorical variables, the majority of which described characteristics of the tackle but also included pre-tackle characteristics (Table 1, Categories for coded variables: Supplementary material). The coding matrix was developed from the templates used in professional rugby union [26, 28–30] and our own work in professional rugby league [22], and in consultation with coaches and researchers familiar with the field of research.

**Table 1:** Propensity for HIAs for tacklers and ball carriers as a function of body position

	HIA events	Propensity (HIAs per 1000 events)	95% CI	Incidence (matches per HIA)
**HIAs as a function of tackler body position**				
Tackler HIAs				
*For upright tackler*	160	1.38	1.19–1.62	2.51
*For bent tackler*	86	0.71	0.58–0.88	4.67
*For falling/diving tackler*	36	0.72	0.52–1.00	11.17
*Total Tackler HIAs*	282	0.99	0.88–1.11	
Ball carrier HIAs				
*For upright tackler*	135	1.17	0.99–1.38	2.98
*For bent tackler*	11	0.09	0.05–0.16	36.55
*For falling/diving tackler*	15	0.3	0.18–0.50	26.80
*Total Ball Carrier HIAs*	161	0.56	0.48–0.66	
**HIAs as a function of ball carrier body position**				
Tackler HIAs				
*For upright ball carrier*	244	1.63	1.44–1.85	1.65
*For bent ball carrier*	8	0.10	0.05–0.20	50.25
*For falling/diving ball carrier*	29	0.53	0.37–0.76	13.86
*Total ball carrier HIAs*	281	0.99	0.88–1.11	
Ball carrier HIAs				
*For upright ball carrier*	121	0.81	0.68–0.97	3.32
*For bent ball carrier*	5	0.06	0.03–0.15	80.40
*For falling/diving ball carrier*	35	0.63	0.46–0.88	11.49
*Total Ball Carrier HIAs*	161	0.56	0.48–0.66	

CI: confidence interval; HIA: head injury assessment

For this study, the focus was on technical elements of the tackler and ball carrier’s actions, and the resultant head impacts. We report the body position of the tackler and ball carrier, head contact of the injured player with the opponent during the tackle, and the evasion method used by the ball carrier. The options coded within each of these categories are summarized in Categories for coded variables (Additional file 1: Table S1).

In addition, a control group of 5694 tackles that did not result in an HIA were coded from 8 randomly selected games from the 2017 and 2018 seasons. Based upon previous research and the requirement for adequate statistical power, we required sufficiently high numbers of control tackles that did not result in head injury. Based upon the typical number of tackles in a rugby league match, and our sample of 472 HIAs, we initially decided that five matches (providing an estimated 3500 total tackles, a seven-to-one ratio for controls to cases) without a head injury event would be sufficient. However, some rare events were not accounted for in these control matches, and so we decided that in order to provide for the denominator required for propensity calculations, we would increase the control set to eight matches. This provided 5694 total tackles. A tackle was defined as ‘any event where one or more tacklers attempted to stop or impede the ball carrier whether or not the ball carrier was brought to ground.’ The control tackles were coded by the same analyst, and they were used to calculate the frequency of each tackle characteristic in normal match play. The analyst has multiple hours of experience coding video footage of professional rugby league and rugby union over many years, as well as multiple years of experience as an in-game, sideline match day staff member identifying potential concussion injuries. This enabled calculation of the propensity of a given tackle scenario to cause an HIA in injuries per 1000 events of each type.

Tackle events were excluded from analysis if (1) the quality of the video footage did not allow the tackle elements to be clearly identified or observed or (2) the video footage was of insufficient quality to apply the coding template to the tackle. In some tackles, only one variable from the coding matrix could not be clearly identified. To ensure larger sample size, these cases were kept in the cohort, and thus the total number of tackles analysed for each tackle characteristic may vary by small amounts, as reported in the results.

### Data Analysis

The event risk or propensity, in HIAs per 1000 tackles for each tackle characteristic, was calculated by dividing the number of HIA events occurring from that tackle characteristic by the total number of that tackle characteristic (obtained from the control cohort) and multiplying by a thousand. The incidence of HIAs was calculated as the period in matches per HIA for each tackle characteristic.

Data are presented as means and 95% CIs. The probability of each tackle characteristic being associated with a player undergoing an HIA was assessed using a Poisson regression with a log link function, using exposure to the characteristic as the offset variable to compare predictor/independent variables. Incident rate ratios (IRRs) were calculated to compare the propensity of two events by expressing the calculated HIA propensity relative to one another. Data were analysed using a standard statistical package (SPSS, Version 24.0), and a conventional type 1 error rate of 0.05 was used, with statistical significance accepted when the 95% CIs did not overlap.

## Results

### Overall Summary

A total of 472 HIAs were identified and coded during the analysis period. Of these, 446 (94.5%) occurred during tackles, with the remaining 26 occurring during open play and off-the-ball collisions. The 446 tackles were explored in detail for subsequent analysis. Overall HIA propensity during tackles was 1.56 HIAs per 1000 tackles (95% CI 1.42–1.71), with a tackle HIA occurring every 0.90 matches (95% CI 0.82–0.99). There were 283 HIAs that occurred to tacklers, who were 1.7 times more likely to experience HIAs than ball carriers (163 HIAs). Tackler propensity was 0.99 tackler HIAs per 1000 tackles (95% CI 0.88–1.11) compared to 0.57 ball carrier HIAs per 1000 tackles (95% CI 0.49–0.66).

### Player Body Position

**Fig. 1:**
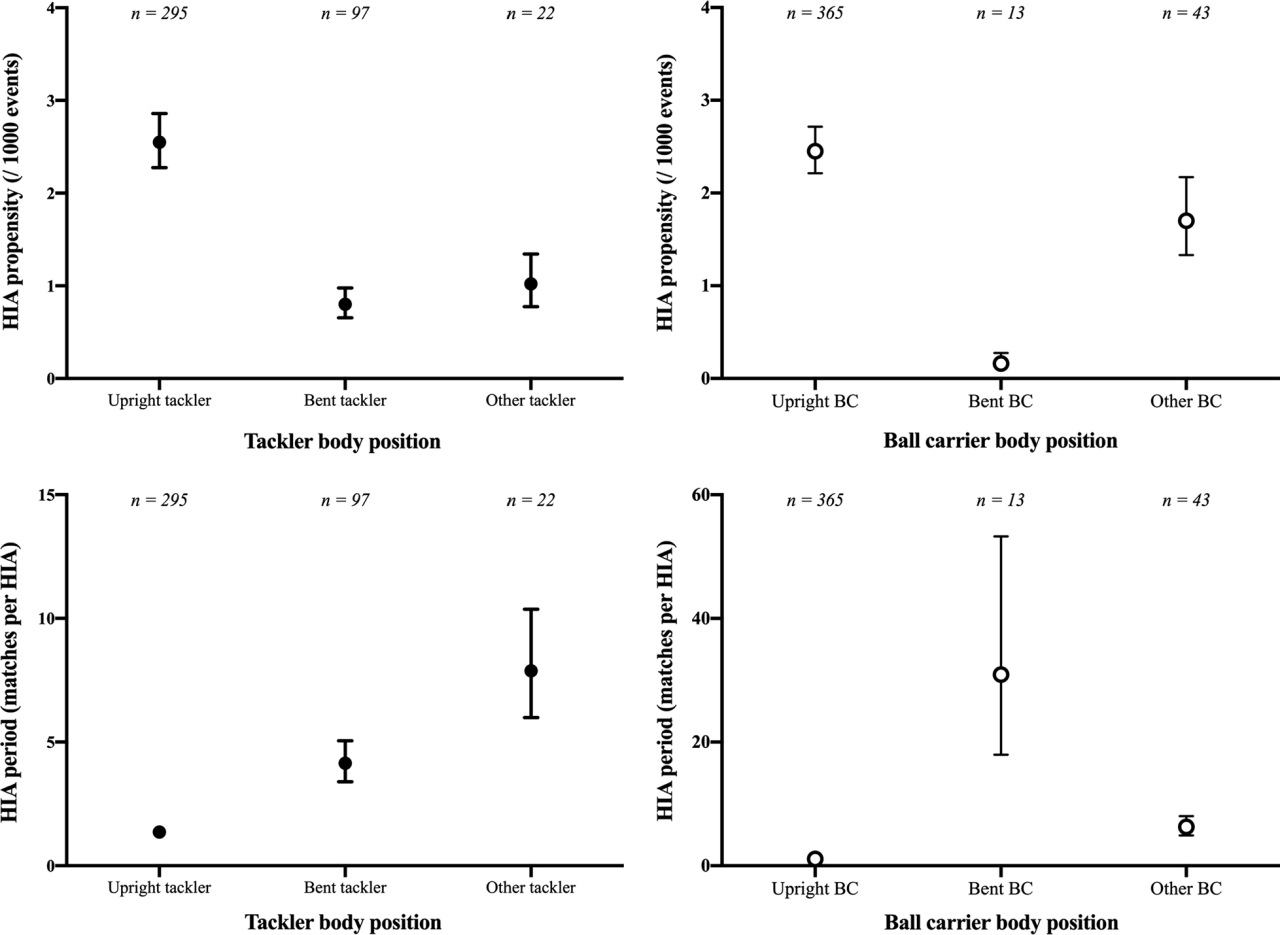
HIA propensity (top) and HIA incidence (bottom) for tackler (left) and ball carrier (right) body positions

The propensity and period for HIAs as a function of player body position is shown in Fig. 1. Three tackles were omitted from the analysis because a tackler body position could not be determined from available video footage. Upright body positions for both tacklers and ball carriers created the greatest risk of HIAs. Upright tacklers were observed in 67% of all tackle HIAs, with an HIA involving an upright tackler every 1.36 matches (95% CI 1.22–1.53) with a propensity of 2.55 HIAs per 1000 tackles (95% CI 2.28–2.96). The propensity for an HIA was 3.2 fold greater when tacklers were upright compared to bent at the waist (0.80 HIAs per 1000 bent tackles, 95% CI 0.66–0.98), a situation that occurred every 4.14 matches (95% CI 3.40–5.06). For falling or diving tacklers, propensity was 1.02 HIAs per 1000 falling/diving tackles (95% CI 0.78–1.34), with a period of 7.88 matches (95% CI 5.99–10.37).

When evaluating risk for various ball carrier body positions, propensity was greatest when ball carriers were upright (2.45 HIAs per 1000 tackles, 95% CI 2.28–2.86), compared to both bent-at-the-waist (0.16 HIAs per 1000 tackles, 95% CI 0.09–0.27) and falling or diving (1.70 HIAs per 1000 tackles, 95% CI 1.33–2.17) ball carriers. HIA incident rates were greatest for upright ball carriers (1.10 matches, 95% CI 0.99–1.22), followed by falling/diving ball carriers (6.28 matches, 95% CI 4.92–8.03) and bent at the waist ball carriers (which were least likely to occur; every 30.92 matches, 95% CI 17.96–53.26).

### Player Body Position and Injured Player

The influence of player body position for HIAs occurring specifically to the tackler and ball carrier are explored in Table 1. When the tackler experienced the HIA (282 HIAs with adequate video footage), 160 (57%) occurred when the tackler was upright. Upright tacklers produce the highest propensity and incidence for tackler HIAs (Table 1), with upright tacklers 1.9 times more likely to experience HIAs than bent tacklers, and 1.4 times more likely to experience HIAs than diving tacklers. When the HIA occurred to the ball carrier (n = 161), the greatest propensity and incidence were also observed for upright tacklers (1.17 ball carrier HIAs per 1000 upright tacklers, 95% CI 0.99–1.34, with a period of 2.98 matches per ball carrier HIA (95% CI 2.52–3.52)).

The propensity for head injury to each player was similarly affected by ball carrier body position. The greatest propensity for an HIA for both tackler and ball carrier occurred when the ball carrier was upright (1.63 tackler HIAs per 1000 upright ball carriers and 0.81 ball carrier HIAs per 1000 upright ball carriers, Table 1).

### Interaction of Tackler and Ball Carrier Body Positions

**Fig. 2:**
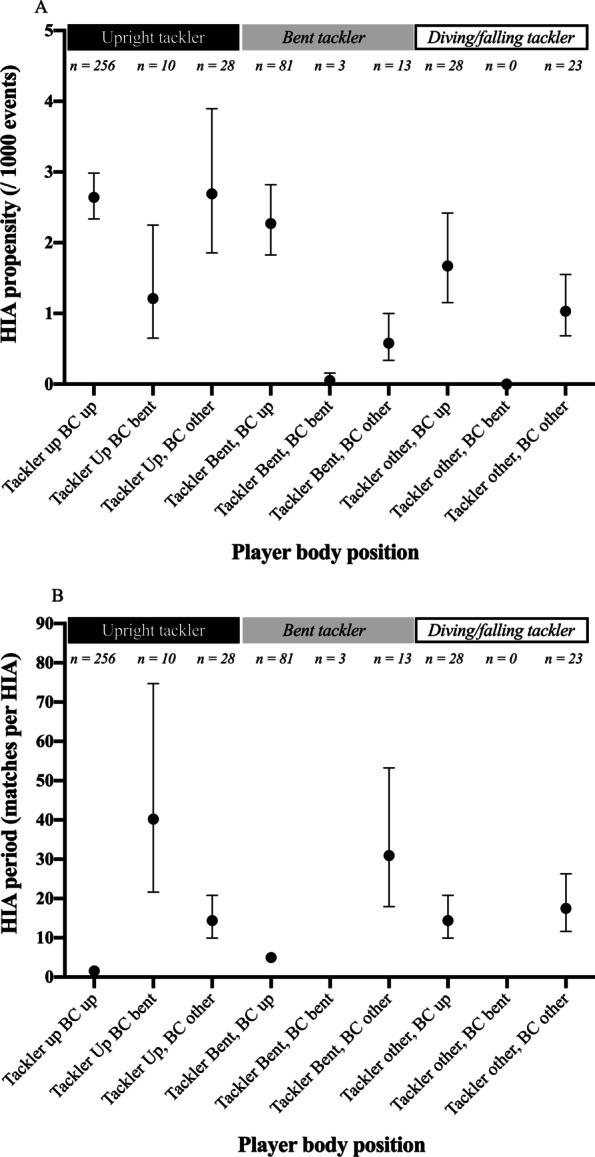
HIA propensity (2A) and incidence (2B) of tackler and ball carrier body position interaction

We next explored how the HIA propensity and incidence were affected by interactions of tackler and ball carrier body position (Fig. 2A). When both players in the tackle were upright, the HIA propensity was 2.64 HIAs per 1000 such tackles (95% CI 2.34–2.98). This was similar to the propensity for tackles where the tackler was upright and the ball carrier was falling/diving (labelled “other” in the figure, 2.69 HIAs per 1000 tackles, 95% CI 1.85–3.90) and tackles with bent tacklers and upright ball carriers (2.27 HIAs per 1000 tackles, 95% CI 1.82–2.82).

The incidence of these highest propensity body position interactions was however different (Fig. 2B). The highest incidence occurred when both tacklers and ball carriers were upright (every 1.57 matches, 95% CI 1.39–1.77). For a bent tackler and upright ball carrier, an HIA occurred every 4.96 matches (95% CI 3.99–6.17), while an upright tackler and falling/diving ball carrier produced an HIA every 14.36 matches (95% CI 9.91–20.79).

The lowest propensity and incidence occurred for tackles where both the tackler and the ball carrier were bent (0.05 HIAs per 1000 tackles, with a period of one every 134 matches). This situation accounted for only three HIAs (< 1% of the total), despite comprising 22% of all tackles in the control cohort (Fig. 2A, note this has been omitted from Fig. 2B for clarity of comparison with more common HIA mechanisms).

### Head contact with opponent

Table 2 shows the HIA propensity and incidence for various head contacts with the opponent in tackles, with tackler and ball carrier HIAs considered separately. The highest propensity for tackler HIAs occurred when the tackler’s head struck the ball carrier’s elbow, boot, or knee, while head contact with the playing surface also resulted in a high risk of tackler HIAs. HIAs from these impacts were however rare, accounting for 25 (9%), 12 (4%), 17 (6%) and 5 (2%) of tackler HIAs, respectively. Their incidence was thus low (Table 2).

**Table 2:** Tackler and Ball Carrier HIAs for different contacts with opponent’s body

	HIA events	Propensity (HIAs per 1000 events)	95% CI	Incidence (matches per event)
**Tackler HIAs as a function of tackler's head contact with ball carrier**				
*Head*	58	10.31	7.96–13.33	6.93
*Shoulder*	66	0.64	0.5–0.81	6.09
*Hip*	47	2.91	2.18–3.87	8.55
*Forearm*	5	0.22	0.09–0.52	80.40
*Elbow*	25	99.50	67.23–147.25	16.08
*Arm*	7	2.14	1.02–4.49	57.43
*Hand*	14	3.36	1.98–5.66	28.71
*Torso*	3	0.03	0.01–0.1	134.00
*Thigh*	8	1.68	0.83–3.35	50.25
*Knee*	17	56.38	35.05–90.7	23.65
*Boot*	12	79.60	45.2–140.16	33.50
*Playing surface*	5	99.50	41.41–239.06	80.40
*Buttocks*	6	10.85	4.87–24.16	67.00
*Back*	8	0.23	0.11–0.45	50.25
*Unidentifiable*	2			
**Ball carrier HIAs as a function of ball carrier's head contact with tackler**				
*Head*	27	5.60	3.83–8.16	14.89
*Shoulder*	49	0.47	0.35–0.62	8.20
*Hip*	3	3.98	1.28–12.34	134.00
*Forearm*	9	8.96	4.65–17.21	44.67
*Elbow*	1	19.90	2.8–141.28	402.00
*Arm*	30	0.91	0.63–1.3	13.40
*Hand*	6	0.17	0.07–0.37	67.00
*Torso*	10	0.10	0.05–0.17	40.20
*Thigh*	2	2.84	0.71–11.36	201.00
*Knee*	5	3.43	1.42–8.24	80.40
*Boot*	1	19.90	2.8–141.28	402.00
*Playing surface*	18			
*Buttocks*	0	0.00		
*Back*	0	0.00		
*Ball*	0	0.00		
*Lower leg*	1			
*Unidentifiable*	1			

CI: confidence interval; HIA: head injury assessment

The highest incidence was found for impacts between the tackler’s head and the ball carrier’s shoulder and head. These head to head and head to shoulder impacts have a relatively low propensity to cause HIAs (0.64 HIAs per 1000 tackles, 95% CI 0.50–0.81 for shoulder) and a moderately high propensity for HIA (10.31 tackler HIAs per 1000 tackles, 95% CI 7.95–13.33 for head), respectively, but because they occur so frequently in the sport, the HIA incidence from these impacts was low (Table 2).

The lowest propensity for HIAs was observed when the tackler’s head contacted the ball carrier’s torso. This situation also resulted in the lowest tackler HIA incidence, with a tackler HIA every 134 matches (95% CI 43.22–415.48, Table 2).

For ball carrier HIAs, propensity was greatest for ball carrier contact with the tackler’s elbow, boot, forearm and head (Table 2), and lowest for contact with the tackler’s torso, hand and shoulder. As was observed for tackler HIAs, the contact types with the highest propensity (elbow, boot and forearm) were rare, accounting for 11 of the 162 ball carrier HIAs (6.8%), while the most common contact types resulting in HIAs were ball carrier contact with the shoulder (49 HIAs, 30.2%), arm (30 HIAs, 18.5%) and head (27 HIAs, 16.7%).

## Discussion

This study explored how the body position of the tackler and ball carrier was associated with the propensity and incidence of HIAs to both players during tackles in the National Rugby League. HIAs occur commonly in the NRL (1.56 HIAs per 1000 tackles, which equates to one tackle-related HIA every 0.90 matches). Our first important finding, consistent with previous research [24–26], is that the tackler is 1.7 times more likely to require a HIA from a tackle than the ball carrier. Of the 446 HIAs, 63% occurred to the tackler. This is a finding similar to that observed in rugby union [24]. This has implications for the injury prevention initiatives designed to reduce risk for HIAs. The application of law typically protects players from the actions of their opponents. However, our data, like that in rugby union, shows that the majority of injuries happen to the tackler.

To explore this further, we focused on the body position of the players in the tackle, because it had previously been shown that an upright tackler creates a greater risk of an HIA than a bent tackler [24]. Our second important finding is to confirm this for rugby league, where the overall propensity for an HIA was 3.2-fold higher when the tackler was upright compared to bent-at-the-waist, and 2.1-fold greater than for a falling/diving tackler (Fig. 1). The bent-at-the-waist position was defined as being hinged at the hip with relatively straight legs, rather than getting into a squat-like position with bent knees. The result is an HIA from upright tacklers every 1.36 matches, compared to one every 4.14 matches for a bent tackler. Because these two body positions account for 83% of all tackles, their relative risks are most important for risk mitigation considerations.

Similarly, we find that HIA propensity and incidence are highest when the ball carrier is upright (Fig. 1). Notably, when we explored the HIA risk to each player in the tackle as a function of their body position, we confirm that the overall risk is greatest when tacklers are upright, but also find that this highest risk exists for both the tackler and the ball carrier specifically (Table 1). Upright tacklers thus create greatest risk to themselves, with an HIA propensity that remains 1.9-fold greater than when the tackler is bent-at-the waist (Table 1), as well as to their opponent ball carrier, whose risk is increased by a factor of 12.8 when the tackler is upright (Table 1).

Naturally, the interaction between the tackler’s body position and that of the body position of ball carriers, is an important factor to consider. Here, we have explored many possible interactions between upright, bent-at-the-waist, and other player positions (comprised of falling/diving, jumping kicking, lying, and slipping). This analysis confirms that when both players are upright the HIA propensity is greater than (1) when both players are bent in the tackle, and (2) when the tackler is upright and the ball carrier is bent. Indeed, for any ball carrier body position, the HIA risk was greatest when the tackler was upright, and for any tackler body position, the HIA risk was lowest when the ball carrier was bent. While the specific interactions did not always reach significance (Fig. 1), the implication is clear, and consistent with what was found in rugby union [24] – the safest body position is when the tackler and ball carrier are bent, and no specific interaction changes this relative risk profile, though certain interactions create similar risk. Overall, however, bent players are considerably less likely to cause head injuries during tackles.

This can be understood when assessing the location of head contact with the opponent that is responsible for causing the HIA. Here, we have examined the HIA risk to the tackler and the ball carrier separately (Table 2). Unsurprisingly, the greatest HIA propensity occurs, generally, for head contact with a hard, bony surface like an elbow, boot, knee or head of the opponent (Table 2). The very highest propensity impacts are however relatively rare, and thus have a low incidence.

Tacklers were more frequently injured by head-to-head (moderate propensity) and head-to-shoulder (low propensity) impacts, while ball carriers were injured by head-to-head, head-to-arm, and head-to-shoulder impacts (Table 2). Collectively, these findings indicate that tackles where the head is very low (knee or below) or very high (head height) create a greater risk, with the safest zone at the level of the opponent’s torso.

The game play risks and their association with HIAs caused by the various head contact locations can be understood when appreciating that player body position exposes players to situations where their heads are more likely to encounter higher risk contact locations. That is, given the high propensity of head-to-head, head-to-elbow, head-to-knee, and head-to-arm impacts to cause HIAs (Table 2), the player body position that creates higher likelihoods of these impact locations is going to produce the highest HIA propensity. This occurs when players are upright, as we have shown (Fig. 1), or very low (diving to the opponent’s knee).

The strategy that may be explored by regulators to reduce injury risk is therefore to drive tackle technique or execution changes that prevent or reduce the likelihood of head-to-head, head-to-elbow, and head-to-knee impacts. Instead, it would be desirable for heads to be in proximity with, and to make contact with, the torso or the shoulder of an opponent in the tackle, because the HIA propensity for these contacts is very low (Table 2). Importantly, even though the HIA incidence from head-to-shoulder impacts is high (period of 6.09 and 8.20 matches for tacklers and ball carriers, respectively), they are low in risk or propensity (Table 2), indicating the interplay between the frequency of an event and its inherent risk. Therefore, if tacklers and ball carriers were to tackle in such a way as to substitute the highest risk head-to-head, head-to-elbow and head-to-knee impacts for impacts with lower risk at the torso or shoulder, the overall number of head injuries during tackles will decrease. This concept, where one behaviour is substituted for another, requires identification of the behaviour with the higher propensity, so that it can be replaced by the identified safer and thus desirable behaviour with the lower propensity.

In this study, we clearly describe a combination of player body positions and head impact locations that span this spectrum from low propensity (bent players, head impacts with the torso of the opponent) to high propensity (upright players and head impacts with opponent’s heads, or diving players and head impacts with opponent’s knees and feet).

Applying this concept to the body position findings we describe previously, it would thus be desirable for tacklers and ball carriers to more often be bent at the waist, with fewer instances where they are upright. This should reduce the overall number of HIAs because the higher risk behaviour (upright players) is substituted with the lower risk behaviour (bent players). It must be cautioned, however, that if the tackler is too low the risk may increase again as a result of more frequent head to knee and head to boot contacts, which we also found to be high in risk, though very rare. Finally, the elbow-to-head scenario we find to have the highest propensity may be reduced through technique training and law interventions to prevent the use of the elbow on opponent’s heads.

The relatively greater propensity for an HIA when players are upright is in part the result of their head proximity to the higher risk body parts of their opponents (heads and elbows, see Table 2), but may also be the result of dynamic elements and biomechanical factors in the tackle that are beyond the scope of this analysis. For instance, it may be that a bent tackler, whose head is in front of their body while their neck is braced, is less susceptible to the neck forces and head accelerations that can cause a concussion [31]. Tierney and colleagues [31] have demonstrated, using a passive biomechanical model, that head and neck kinematics and mechanics are significantly affected by the area of contact, with higher linear and angular acceleration of the head for ball carrier during upright, higher contacts [31]. Such a phenomenon may contribute to our findings, and may be further moderated by the relative head and neck position of each player when bent at the waist. Also, the context of the tackle may change, with elements of speed, acceleration, direction and tackle technique altered when players are bent compared to upright.

The present study explored mechanisms of head injury risk during tackles for various tackler and ball carrier body positions and head proximities. It must also be considered that tackle proficiency is a crucial determinant of head injury risk, as has been previously documented [32–34]. Within each of the higher risk scenarios we describe here (upright tacklers, heads in proximity with one another), there may be technical elements of tackle execution, described in that research, that mitigate or increase the risk we describe, and interventions targeting reductions in head injury risk should seek to combine the findings of the present study with coaching interventions, led by such research, to address all head injury risk factors.

### The Biomechanical Approach to Reducing the Ball Carrier’s Height

Ball carriers’ trunk posture will alter their stability via the location of their centre of gravity within (a) the base of support and (b) via the principle of moments. That is, with a more bent at waist posture the location of the centre of gravity will move more anterior within the base of support and enable a longer displacement for the centre of gravity to travel before it moves outside the base of support and the ball carrier becomes unstable. The principle of moments relates to the vertical location of the centre of gravity and the application of the external force by the tackler. If the ball carrier lowers his vertical location of the centre of gravity, it decreases the moment arm (i.e. the distance from the application of the external force by the tackler to the ground). So, by the ball carrier adopting a bent at waist posture, compared to an upright posture, a smaller magnitude of moment is created due to the smaller moment arm when the vertical location of centre of gravity is closer to the ground.

### Performance Considerations

The value of safety recommendations, and the successful implementation (acceptance and uptake) of an intervention, can be highly contingent upon performance considerations [35, 36]. Any recommendation that reduces the risk of an injury, but adversely affects performance, is highly unlikely to be implemented by players or coaching staff [37]. In this context, one of the main objectives of a tackler is to smother the ball and the ball carrier to prevent an offload. Tackle techniques that both reduce the height at which the tackler contacts the ball carrier and still enables the tackle strategy to ‘wrap up the ball’ will be crucial for addressing injury risk and performance. For ball carriers, reducing their height entering contact with the tackler is likely to adversely affect their performance in terms of attaining/maintaining their maximum sprinting speed in their attempt to gain territory down the field, or executing an off-load, so consideration regarding the degree to which the height could be reduced, to reduce the risk of injury, but not adversely affect performance, requires further investigation [38]. Reducing the ball carrier’s height can be achieved either by trunk lean, hip flexion, or knee flexion, or a combination of all three. Investigating the extent to which each of these factors individually, or in combination, are successful in reducing injury risk and not adversely affecting performance requires the measurement of the centre of mass location and all three angles.

### Limitations

There are a number of limitations in the current study. First, the interpretation and coding of the tackle variables are subjective. All events were coded by one analyst to avoid any considerations of between-rater differences, though the subjective nature represents a potential source of error in the analysis. The present method of analysis employs a discreet approach to identify specific tackle characteristics that may be the target of risk mitigation strategies. The characteristics do however interact with one another, and while in this study we have attempted to explore these interactions for body position, head contact and ball carrier evasion method, the tackle is a dynamic and complex event during which many factors may affect risk in subtle ways. Second, a number of the tackle variables occurred rarely, leading to sparse data, and should be interpreted with caution. Third, this analysis quantified the propensity of an HIA in the tackle, as a function of the numerous variables (e.g. body position, speed, head proximity, tackle type, etc.) of the first tackler and the ball carrier. The initial contact in the tackle has been shown to be the cause for most HIAs in the NRL (455/727 HIAs, 62.6% from the 2018, 2019, and 2020 NRL seasons). Rugby league tackles often involve multiple tacklers, and contact may occur simultaneously; the current study has not considered the specific behaviours of the later-arriving players to the tackle event injury risk. Only the overall tackle risk as a function of the first contact in the tackle is discussed here. Fourth, the results may not be generalizable to other levels of play or women players, and future research may replicate the current study at the sub-elite level and in women’s rugby league.

## Summary and Conclusions

We show that the tackler is more likely to sustain a head injury than the ball carrier, and that this risk is greatest when both players are upright, both to the tackler and the ball carrier. Risk is lowest when players are bent in the tackle, the result of which is that head contact is more likely to occur between a player’s head and the torso and shoulder of an opponent, rather than high risk and frequency head-to-head contacts, and high-risk head-to-knee and head-to-boot impacts. We find that this bent position is generally safer for any head contact and any ball carrier evasion method, with few circumstances where being upright reduces the risk compared to being bent at the waist. The implication is that risk mitigation that brings players’ heads out of high-propensity locations that occur when tackling too high or too low may contribute to a reduction in overall head injury risk in Rugby League. This can be achieved in large part by preventing upright tackles that result in head-to-head proximity and impacts, aiming instead for a slight lowering of height to produce head-to-torso contacts and proximities, while still emphasizing the risk of excessively low tackles that increase the likelihood of head to knee and boot impacts. Tackle technique education and training to achieve these desired outcomes given the dynamic and complex nature of the sport could help reduce head injuries.

### How Might It Impact on Clinical Practice in the Future?

This study replicates in rugby league prior findings from rugby union relating to tackle characteristics and concussion risk. Rugby union has introduced intervention strategies, through rule modifications, to try to reduce concussion risk. It might be useful to explore sanction reinforcement and change, as well as coaching methods for reducing the occurrence of upright tackles, which are shown to result in a high proportion of head injuries.

## Supplementary Information

**Additional file 1**. Categories for coded variables.

## Data Availability

The statistical analyses and underlying data supporting the conclusions of this article will be made available by the authors to qualified researchers for research purposes, without undue reservation.

## Abbreviations

HIA: Head Injury Assessment
NRL: National Rugby League
SIS: Sideline Injury System
