# Supplementary material for: A Case–Control Study of Tackle-Based Head Injury Assessment (HIA) Risk Factors in the National Rugby League
Source: Sports Med Open. 2021 Nov 17;7:84. doi: 10.1186/s40798-021-00377-9 (PMC8599744; doi:10.1186/s40798-021-00377-9)
Supplement: Supplementary file 1 — Additional file 1. Categories for coded variables. [file 40798_2021_377_MOESM1_ESM.docx]

Supplementary Table 1. Categories for coded variables.

| The tackler and ball carrier characteristics explored in the present study are summarized below | |
| --- | --- |
| Tackler and BC body positions | |
| Upright | The player is standing in an upright position, with the knees only slightly bent and with minimal hip flexion |
| Bent at the waist | The player is bent at the waist or crouched |
| Falling/diving | The player is in the process of diving or falling to ground at the point of contact |
| Contact body part | |
| For all the below body parts, the head of the player is in proximity to, or makes contact with, the indicated body part of the opponent, or the ground. | |
| Head  Shoulder  Hip  Forearm  Elbow  Arm  Hand  Torso  Thigh  Knee  Boot  Playing surface  Buttocks  Back  Ball  Lower leg  Goal post | |
| The remaining tackle characteristics were coded but are not explored in the current study, but will be described in future research | |
| Player position | |
| Time of HIA | |
| Tackle number in set | |
| Speed of defensive line | |
| Tackle outcome | |
| Accidental head contact with team-mate | |
| Number of tacklers involved | |
| Foul play | |
| Anticipation of contact by player | |
| Height and weight differential between players | |
| Tackle direction | |
| Evasive action of the ball carrier | |
| Ball carrier evasion method | |
